# Maternal serum ferritin across gestation and risk of small-for-gestational-age: a longitudinal cohort study

**DOI:** 10.3389/fnut.2026.1766451

**Published:** 2026-04-24

**Authors:** Nana Guo, Huiqin Mo, Wenting Zhou, Xiaohua Liu

**Affiliations:** 1 Department of Obstetrics, Shanghai Key Laboratory of Maternal Fetal Medicine, Shanghai First Maternity and Infant Hospital, School of Medicine, Tongji University, Shanghai, China; 2 Department of Obstetrics and Gynecology, The Seventh People's Hospital of Shanghai University of Traditional Chinese Medicine, Shanghai, China

**Keywords:** iron supplementation, longitudinal changes, pregnant women, serum ferritin, small-for-gestational-age

## Abstract

**Background:**

Iron supplementation is essential for maintaining maternal-fetal health during pregnancy. However, research on the relationship between serum ferritin (SF) and small-for-gestational-age (SGA) births remains limited and inconclusive.

**Objective:**

This study aimed to investigate the association between maternal SF levels and SGA during pregnancy.

**Methods:**

We utilized electronic medical records from women who delivered at Shanghai First Maternity and Infant Hospital between 2018 and 2020. Maternal SF levels were measured at 8.0–13.6 weeks' gestation (GW) and 29.0–31.6 GW. Based on these measurements, participants were categorized into three groups according to SF concentration percentiles: low (< 25th), medium (25th to < 75th), and high (≥75th). The association between maternal SF levels and SGA was evaluated using binary logistic regression. Additionally, a restricted cubic spline model was employed to explore potential nonlinear relationships between SF levels and the risk of SGA.

**Results:**

A total of 17,451 pregnant women were included. At 29.0–31.6 weeks of gestation, women with elevated serum ferritin (SF) (≥18.1 ng/mL, ≥75th percentile) had a significantly higher incidence of SGA than those with lower levels [4.4% vs. 2.4%; adjusted odds ratio (aOR) = 1.418, 95% CI: 1.089–1.846, *P* = 0.009]. A significant positive dose-response relationship between SF levels and SGA risk was observed (*P* for trend < 0.0001), with a threshold of 12.1 ng/ml. Compared with women with consistently low SF levels in both trimesters, the risk of SGA was significantly increased in two groups: 1) those with medium first-trimester SF (31.5 to < 81.9 ng/ml) and high third-trimester SF (≥18.1 ng/ml) (aOR = 1.712, 95% CI: 1.138–2.576, *P* = 0.01); and 2) those with high SF in both trimesters (first: ≥81.9 ng/ml; third: ≥18.1 ng/ml) (aOR = 1.676, 95% CI: 1.118–2.513, *P* = 0.012). However, SF levels dropping from high to medium between trimesters was not associated with an increased risk of SGA (aOR = 0.951, 95% CI: 0.614–1.472).

**Conclusions:**

Our findings demonstrate an independent and positive association between elevated maternal serum ferritin levels during the third trimester of pregnancy and increased risk of SGA. These results challenge the current practice of routine iron supplementation in pregnant women with normal iron levels.

## 1 Introduction

Small-for-gestational-age (SGA) was defined as the birth weight below the 10th percentile for gestational age or less than −2 standard deviations (−2 SD) of the mean birthweight for the same gestational age (1). SGA neonates have higher mortality, shorter future height, and are more susceptible to obesity, metabolic disease, accelerated aging, and coronary vascular disease (2–4), as well as lower IQ during childhood (5). The etiology of SGA encompasses maternal and obstetric factors, placental insufficiency, and fetal genetic factors. Significant gaps persist in understanding the etiology of SGA and developing effective interventions. A study suggests that mothers with low hemoglobin levels in late pregnancy are more likely to have low birth weight infants (6). In contrast, Yun Tao et al. found a linear positive correlation between maternal ferritin level and risk of adverse birth weight outcomes, based on a sample size of 3,566 cases (7). Recent prospective studies indicate that iron supplementation in non-anemic pregnant women is associated with an increased risk of low birth weight, especially at higher doses (8, 9). Peña-Rosas et al. noted in their 2012 review that daily iron supplementation might reduce the risk of low birth weight in infants; however, this conclusion was removed the subsequent updates (2015 and 2024) (10–12).

According to the World Health Organization (WHO) 2019 report, 29.9% of women of reproductive age have anemia, and the prevalence among pregnant women is 36.5% (13). Recognizing the adverse impacts of anemia on maternal health and fetal development, the WHO and United Nations International Children's Emergency Fund (UNICEF) have proposed reducing the prevalence of anemia by 50% among women of reproductive age (15–49) by 2030, in line with the United Nations Sustainable Development Goals (14). Driven by this goal, public awareness of iron supplementation has gradually increased, which paradoxically has led to iron overload. A growing body of evidence indicates that iron overload is associated with an increased risk of cardiovascular diseases, neurodegeneration, infections, and malignancies in humans (15–18). In obstetrics, studies have similarly revealed a positive correlation between maternal ferritin levels and the incidence of adverse pregnancy outcomes, such as gestational diabetes (GDM), preeclampsia (PE), and preterm birth (19, 20). Findings from our previous studies on gestational diabetes and hypertensive disorders of pregnancy have further corroborated this association (21, 22).

However, studies on the association between SF levels and SGA remain limited and inconsistent. Therefore, the present study aimed to evaluate the association between maternal serum ferritin levels and the risk of SGA in a longitudinal cohort.

## 2 Methods

### 2.1 Data source and study participants

This retrospective cohort study was conducted at Shanghai First Maternity and Infant Hospital (SFMIH), Tongji University School of Medicine. Childbirth data were extracted from the hospital's electronic medical records system for the period 2018 to 2020. The study was approved by the SFMIH Ethics Review Board (Approval No.: KS21270).

The study included participants who underwent SF concentration assessments at 8.0–13.6 and 29.0–31.6 gestational weeks. Following the exclusion of women with multiple gestation, stillbirth, chronic infections, cardiac disease, renal disorders, and other conditions that adversely affect SF levels during pregnancy, a total of 17,451 women met the inclusion criteria and were included in the final analysis. A flowchart of the study population is presented in Supplemental Figure 1.

The analysis was adjusted for the following potential confounders: maternal age, pre-pregnancy body mass index (BMI), parity, gestational age at delivery, and pregnancy complications including PE, premature rupture of membranes (PROM), and preterm birth. White blood cell (WBC) count and hemoglobin (Hb) levels during pregnancy were also included in the model. Pre-pregnancy BMI was calculated based on self-reported pre-pregnancy weight and height. Gestational age were estimated from the self-reported last menstrual period and confirmed by first-trimester ultrasound.

Maternal serum ferritin levels were measured during early (8.0–13.6 weeks) and late (29.0–31.6 weeks) gestation using a commercial Maglumi ferritin immunoluminometric assay on a Beckman Coulter DXL 800 analyzer in the hospital laboratory. All reagents and calibrators were obtained from the manufacturer, with an interassay coefficient of variation < 5% (23).

In accordance with international guidelines, small-for-gestational-age (SGA) was defined as a birth weight either below the 10th percentile or less than −2 standard deviations (−2 SD) from the gestational age-specific mean (24).

### 2.2 Statistical analysis

Missing values [BMI: 975 cases (5.6%); early-/late-pregnancy platelet counts: 2 and 1 case] were imputed by regression analysis using SPSS. No statistically significant difference was found in BMI before vs. after imputation (before: *n* = 16,476, 21.74 ± 3.00; after: *n* = 17,451, 21.75 ± 2.99; independent samples *t*-test: *t* = −1.43, *P* = 0.886, Cohen's *d* = −0.002). Normally distributed variables were expressed as mean ± standard deviation (SD), while non-normally distributed variables were presented as median (minimum–maximum). We applied: (1) the χ^2^ test for categorical variables, (2) the independent samples *t*-test for normally distributed continuous variables, and (3) the Wilcoxon rank-sum test for non-normally distributed continuous variables. Based on the distribution, we categorized SF concentration into three levels using percentile cut-offs: low (< 25th percentile), medium (25th−75th percentile) and high (≥75th percentile). The stratification allowed examination of the SF-SGA association across different gestational periods. Maternal SF levels were measured during early (8.0–13.6 weeks) and late (29.0–31.6 weeks) gestation, with levels at each stage classified as low, medium, or high. The longitudinal association between these SF categories and SGA risk was assessed using a 3 × 3 table, which displayed all possible pairs (e.g., low-low, low-medium,..., high-high).

Odds ratios (ORs) with 95% confidence intervals (CIs) were calculated using binary logistic regression analysis. Furthermore, adjusted odds ratios (aORs) were calculated after adjusting for potential confounders: maternal age, pre-pregnancy BMI, parity, PE, PROM, preterm birth, WBC counts and Hb levels. A restricted cubic spline model with 4 knots was employed to explore the potential linear dose-response relationship between serum ferritin levels and SGA risk using RStudio 4.4.3, with adjustment for relevant covariates. All other analyses were performed using SPSS (version 27; IBM Corp., Armonk, NY, USA). All statistical tests were two-tailed, with statistical significance set at *P* < 0.05.

## 3 Results

A total of 17,451 participants met the eligibility criteria. Table 1 summarizes the baseline characteristics of SGA and non-SGA cases. Women in the SGA group were younger, thinner, and were more likely to be primiparous. They had a lower incidence of PROM and lower first-trimester WBC counts and Hb levels compared to the non-SGA group. However, they had a higher incidence of PE and preterm birth. Notably, while their hemoglobin levels were lower in the first trimester, they were higher at 29.0–31.6 GW.

**Table 1:** Baseline and obstetric characteristics of SGA.

Variable	SGA (*n* = 536)	Non-SGA (*n* = 16,915)	*P*
Age, year			
< 35 (*n*, %)	460 (85.8)	13,653 (80.7)	0.003
≥35 (*n*, %)	76 (14.2)	3,262 (19.3)	
Pre-pregnancy BMI, kg/m^2^			
< 18.5 (*n*, %)	109 (20.3)	1,583 (9.4)	< 0.001
18.5–24 (*n*, %)	366 (68.3)	11,878 (70.2)	
24–28 (*n*, %)	54 (10.1)	2,779 (16.4)	
≥28 (*n*, %)	7 (1.3)	675 (4.0)	
Parity (*n*, %)			
= 1	480 (89.6)	12,770 (75.5)	< 0.001
≥3	2 (0.4)	155 (0.9)	0.246
Preeclampsia (*n*, %)	47 (8.8)	513 (3.0)	< 0.01
Gestational diabetes (*n*, %)	73 (13.6)	2,072 (12.2)	0.342
Intrahepatic cholestasis in pregnancy (*n*, %)	9 (1.7)	193 (1.1)	0.252
Premature rupture of membranes (*n*, %)	72 (13.4)	2,964 (17.5)	0.014
Preterm (*n*, %)	34 (6.3)	711 (4.2)	0.016
Cesarean delivery (*n*, %)	219 (40.9)	7,489 (44.3)	0.117
Apgar scores < 8 (*n*, %)	5 (0.9)	119 (0.7)	0.435
Gestational week at first collection, week^b^	11.3 (8–13.6)	11.2 (8–13.6)	0.492
Gestational week at second collection, week^b^	31.0 (29–31.6)	31.1 (29–31.6)	0.078
White blood cell, 10^9^/L^a^			
At first collection	8.10 ± 1.86	8.36 ± 1.95	0.002
At second collection	9.02 ± 1.98	9.12 ± 2.04	0.292
Hemoglobin, g/L^a^			
At first collection	126.09 ± 8.86	126.90 ± 8.99	0.039
at second collection	117.12 ± 9.30	115.38 ± 9.44	< 0.001
Platelet, 10^9^/L^a^			
At first collection	225.82 ±5 1.78	229.74 ±5 0.21	0.075
At second collection	207.98 ± 51.02	205.16 ± 49.46	0.194

BMI, body mass index; SGA, small-for-gestational-age.

^a^Values are means ± SDs.

^b^Values are medians (minimum value-maximum value).

As presented in Table 2, serum ferritin concentration was divided into three groups based on the 25th (31.5 ng/ml at 8.0–13.6 GW, 7.5 ng/ml at 29.0–31.6 GW) and 75th (81.9 ng/ml at 8.0–13.6 GW, 18.1 ng/ml at 29.0–31.6 GW) percentiles. The low-level group served as the reference. A higher incidence of SGA was observed with elevated SF levels at 29.0–31.6 GW (4.4% vs. 2.4%). In multivariable-adjusted models adjusting for maternal age, pre-pregnancy BMI, parity, PE, PROM, preterm birth, WBC counts, Hb levels, the high-level group had 1.4-fold higher odds of SGA compared to the low-level group in the third trimester (adjusted OR = 1.418, 95% CI: 1.089–1.846, *P* = 0.009). This association was not statistically significant in the first trimester (8.0–13.6 GW) (3.5% vs. 2.6%, adjusted OR = 1.156, 95% CI: 0.899–1.486, *P* = 0.258).

**Table 2:** Associations of SF level with SGA risk.

Variables	SGA (*n*, %)	Unadjusted ^a^OR (95% CI)	Adjusted ^b^OR (95% CI)	*p*
Serum ferritin levels at 8.0–13.6 GW, ng/ml				0.510
Low level (< 31.5)	114 (2.6)	1	1	
Medium level (31.5–81.9)	269 (3.1)	1.181 (0.946–1.475)	1.112 (0.887–1.394)	0.356
High level (≥81.9)	153 (3.5)	1.349 (1.054–1.725)	1.156 (0.899–1.486)	0.258
Serum ferritin levels at 29–31.6 GW, ng/ml				< 0.001
Low level (< 7.5)	105 (2.4)	1	1	
Medium level (7.5–18.1)	240 (2.7)	1.137 (0.901–1.434)	0.907 (0.709–1.161)	0.440
High level (≥18.1)	191 (4.4)	1.832 (1.438–2.333)	1.418(1.089–1.846)	0.009

SGA, small-for-gestational-age; GW, gestation weeks; SF, serum ferritin.

^a^Odds ratios (ORs) with 95% confidence intervals (CIs) were derived from binary logistic regression.

^b^Adjusted for: maternal age (dichotomized), body mass index (BMI, categorized as an ordinal variable), parity (dichotomized), PE (yes/no), PROM (yes/no), and preterm (yes/no), white blood cell count (continuous), and hemoglobin levels (continuous) at measurement.

**Figure 1:**
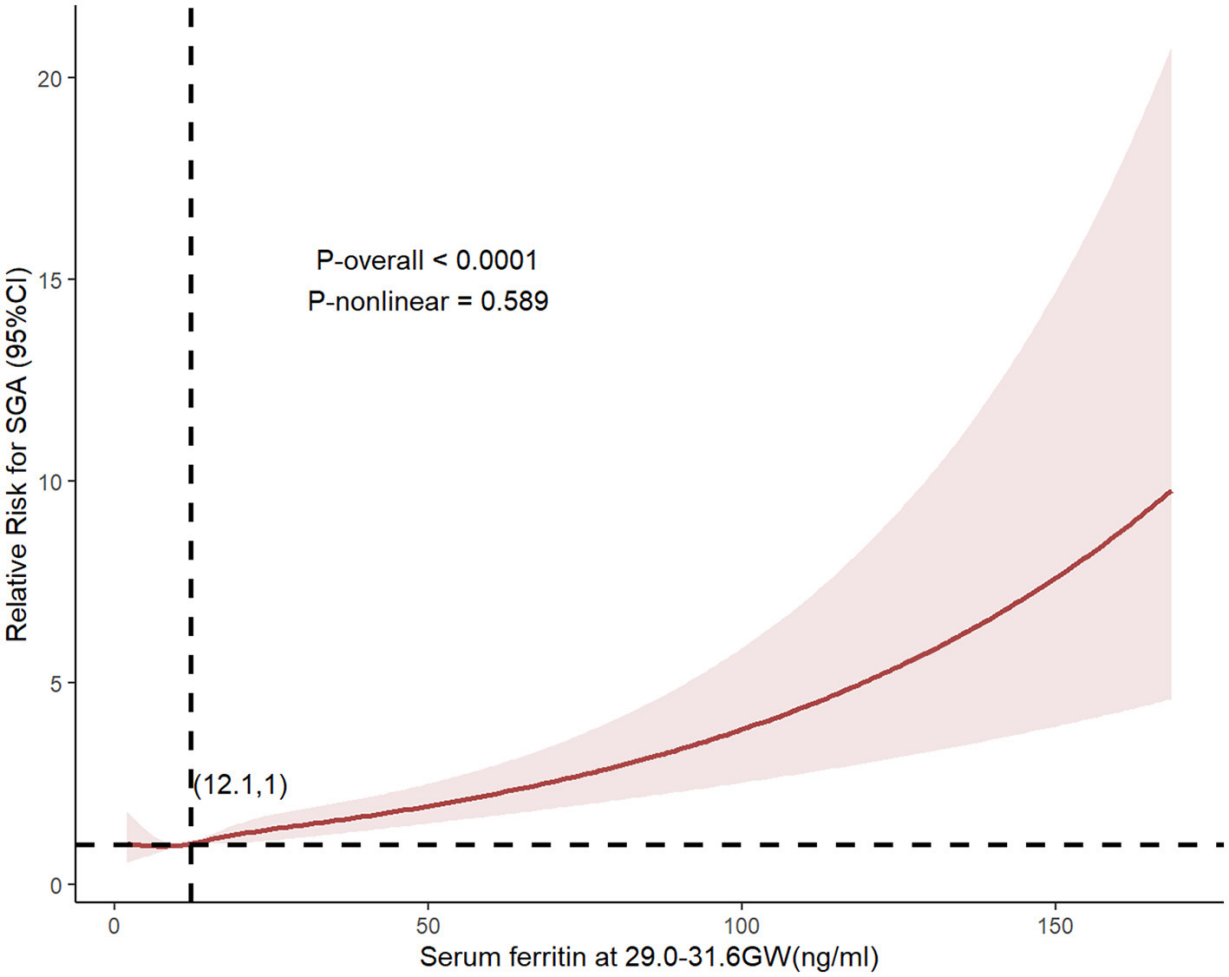
Dose–response relationships between maternal serum ferritin (SF) concentrations and small-for-gestational-age (SGA) risk. The solid line represents the point estimates of adjusted odds ratios (aORs), with dashed lines indicating 95% confidence intervals (CIs). These associations were derived from binary logistic regression models adjusted for: maternal age (continuous), BMI (continuous), parity (categorized as an ordinal variable), PE (yes/no) and PROM (yes/no), and preterm (yes/no), WBC count (continuous) and hemoglobin concentration (continuous).

Restricted cubic spline (RCS) analysis presented in Figure 1 revealed a significant dose-response relationship between third-trimester SF levels and SGA risk (*P*_overall_ < 0.0001). Although the nonlinearity test was not statistically significant (*P* = 0.589), the RCS curve demonstrated a threshold at 12.1 ng/mL (aOR = 1.021, 95% CI: 1.003–1.040), after which a dose-dependent relationship was observed.

We conducted a longitudinal cohort study with serial measurements at 8.0–13.6 and 29.0–31.6 GW to investigate the impact of longitudinal maternal SF level changes on SGA risk. As shown in Table 3, the low-low group was designated as the reference group, against which all other groups showed a higher SGA risk. Two distinct trajectories were found to be significantly associated with an increased risk of SGA. Women who maintained high SF levels in both trimesters (high-high group) had significantly higher odds of SGA (adjusted OR = 1.676, 95% CI: 1.118–2.513, *P* = 0.012). Similarly, a trajectory from medium SF in the first trimester to high SF in the third trimester (medium-high group) was also associated with a substantially increased risk (adjusted OR = 1.712, 95% CI: 1.138–2.576, *P* = 0.01). However, no increased SGA risk was observed for women whose SF level decreased from high to medium (adjusted OR = 0.951, 95% CI: 0.614–1.472, *P* > 0.05). Moreover, iron supplementation did not increase the incidence of SGA among women with low SF levels in the first trimester (low-high group: adjusted OR = 1.194, 95% CI: 0.643–2.218, *P* > 0.05).

**Table 3:** The longitudinal investigation of serum ferritin levels on the risk of SGA.

Groups of SF level (first-third trimesters)	SGA (*n* %)	Adjusted Oa (95% CI)	*P*
Low-low	37 (2.0%)	1	
Low-medium	61 (3.0%)	1.185 (0.773–1.816)	0.437
Low-high	16 (3.4%)	1.194 (0.643–2.218)	0.575
Medium-low	57 (2.7%)	1.279 (0.838–1.951)	0.254
Medium-medium	126 (2.7%)	1.035 (0.706–1.516)	0.862
Medium-high	86 (4.5%)	1.712 (1.138–2.576)	0.01
High-low	11 (2.8%)	1.194 (0.601–2.375)	0.612
High-medium	53 (2.7%)	0.951 (0.614–1.472)	0.821
High-high	89 (4.5%)	1.676 (1.118–2.513)	0.012

SGA, small-for-gestational-age; SF, serum ferritin.

^a^Binary logistic regression models were adjusted for maternal age (dichotomized), PE (yes/no) and PROM (yes/no), and gestational age at delivery (preterm/term), WBC count (continuous), and hemoglobin concentration (continuous).

## 4 Discussion

Our longitudinal cohort study yielded four key findings: (1) Elevated maternal serum ferritin levels in the third trimester were positively associated with an increased risk of SGA; (2) A significant positive association was observed between serum ferritin levels ≥12.1 ng/ml at 29–31.6 GW and SGA risk; (3) Women with iron-replete SF levels in the first trimester that remained high in the third trimester had an increased risk of SGA; (4) The risk of SGA returned to baseline in women whose SF levels decreased from high in the first trimester to lower levels during gestation.

Alwan et al. conducted a study of 362 women in Leeds, UK, which found that first-trimester iron depletion (SF < 15 μg/L) was associated with a higher risk of SGA. However, this association was no longer significant after adjusting for Hb (adjusted OR = 1.6; 95% CI: 0.8, 3.2) (23). In a retrospective study of 2,327 pregnant women in Guangdong Province, China (2015–2020), Yang L et al. found that each SD increase in SF concentration at 16–18 weeks of gestation was associated with a 21% increase in the risk of SGA (aOR = 1.21; 95% CI: 1.06, 1.38; *P* = 0.032) (24). Similarly, Tao Y et al. reported a linear correlation, with stratified analysis revealing an even stronger association for severe SGA (aOR = 2.30; 95% CI: 1.47–3.61) (7). Our study supplemented the data for both the first and third trimesters. In an analysis of three recent trials from Ghana, Malawi, Bangladesh, Kathryn G. Dewey and colleagues found that a combination of higher third-trimester iron status and lower sTfR concentrations was associated with smaller birth size (25). Analysis of our large-scale cohort demonstrated a positive correlation between SF levels and SGA risk, especially in the third trimester. Some researchers have conducted prospective studies on iron supplementation in non-anemic pregnant women, showing that those treated with iron, particularly at higher doses, had an increased risk of delivering low birth weight infants (8, 9). Using a longitudinal cohort design, we observed no significant increase in SGA risk for women with low SF levels in the first trimester who elevated to high levels by the third trimester, which confirmed the view that the timing and pattern of ferritin elevation are critical to the development of SGA.

Several studies have explored the potential mechanisms linking iron overload to the pathogenesis of SGA. Prenatal iron supplementation raises maternal hemoglobin concentrations, which might impair fetal development by affecting placental angiogenesis (26). Placental iron transport occurs through a directional transcellular pathway, as maternal and fetal blood does not mix and iron transporters exhibit polarized distribution on syncytiotrophoblast (STB) membranes (27–29). Higher maternal serum ferritin levels may affect fetal weight by several mechanisms: (1) Dysregulation of the Hepcidin-ferroportin axis, the key regulator of iron transport balance (30–32). Research on preeclampsia revealed elevated hepcidin expression and related gene upregulation in fetal growth restriction (FGR) placentas compared with normal controls (33). (2) Ferroptosis, which is characterized by non-apoptotic, iron-dependent accumulation of lethal reactive oxygen species (ROS) in lipid membranes (34). Compared with appropriate for gestational age (AGA) controls, MDA levels (a biomarker of oxidative stress) are significantly elevated in both mothers and SGA infants during delivery. Notably, umbilical vein MDA concentrations exceed arterial levels in SGA cases (35). Consistent with these findings, SGA neonates exhibit higher MDA levels than AGA neonates (36). Acute atherosclerotic changes caused by ferroptosis have been identified in the placenta maternal vascular walls among preeclampsia cases complicated by SGA infants (37, 38). A study on pregnant mice showed that excessive iron during pregnancy may trigger inflammatory diseases caused by liver macrophage dysfunction, leading to fetal growth restriction and potentially more severe pregnancy complications, such as fetal demise (39). While our study links elevated SF levels to SGA risk, the underlying mechanisms require further investigation.

Currently, there is no unified diagnostic threshold for iron deficiency using serum ferritin (SF) in pregnancy. The WHO recommends an SF level < 15 ng/ml in early pregnancy as indicative of iron deficiency (13), while the United States, the United Kingdom, and Australia suggest < 30 ng/ml (40–42). In China, a threshold of < 20 ng/ml is recommended (43), although related high-quality research data are extremely limited. In clinical practice, Chinese clinicians advise iron supplementation for pregnant women with SF levels below 30 ng/ml (43). The increase in plasma volume during the second trimester and changes in inflammatory markers in the third trimester make it difficult to establish a single, fixed ferritin concentration for diagnosis. In our study, maternal serum ferritin levels decreased markedly from the first to the third trimester, with the median dropping from 51.7 ng/ml to 11.3 ng/ml. Consequently, the prevalence of SF levels < 30 ng/ml increased from 23% to 94%. However, only 25% of these women were anemic at 29.0–31.6 weeks of gestation. A growing body of research is raising concerns about the dangers of excessive iron supplementation during pregnancy (44). To account for the dynamic changes in ferritin concentrations across pregnancy, it is imperative to establish trimester-specific SF reference ranges.

Our study has several limitations. Firstly, while serum ferritin (SF) is a widely used biomarker for iron status, it is also an acute-phase reactant that increases during inflammation, reflecting the degree of acute and chronic inflammatory conditions (45, 46). Patients with such inflammatory diseases often develop anemia of inflammation, characterized by reduced iron availability, inhibited erythropoiesis, and shortened red blood cell lifespan. Typically, SF levels in anemia of inflammation exceed 100 ng/ml (47). Although inflammatory markers like C-reactive protein (CRP) or α-1-acid glycoprotein (AGP) could help adjust for inflammatory confounding (46), these data were unavailable in our retrospective study. To mitigate this limitation, we excluded participants with anemia of inflammation, defined as having SF >100 ng/ml and Hb < 110 g/L. Concurrently, we adjusted for white blood cell (WBC) count as a proxy for systemic inflammation, along with other potential confounders including maternal age, preeclampsia (PE), premature rupture of membranes (PROM), gestational age at delivery, and hemoglobin (Hb) levels. Therefore, future studies should incorporate additional parameters, such as specific inflammatory markers, to enable a more robust analysis. Secondly, due to the retrospective design, we lacked precise data on iron supplementation (e.g., dose, duration). We therefore used longitudinal changes in maternal SF levels across gestation, which indirectly reflect iron supplementation status. Thirdly, the retrospective design inherently limits the causal interpretation of the association between SF and SGA. Further prospective cohort studies are needed to establish causality.

In conclusion, our study demonstrates a significant positive association between SF levels and SGA risk among pregnant women in the third trimester, highlighting the importance of monitoring maternal iron status during pregnancy. Considering the increased risk of SGA, routine iron supplementation for pregnant women with iron repletion is inappropriate.

## Data Availability

The original contributions presented in the study are included in the article/Supplementary material, further inquiries can be directed to the corresponding authors.
