# Supplementary material for: Maternal serum ferritin across gestation and risk of small-for-gestational-age: a longitudinal cohort study
Source: Front Nutr. 2026 Apr 24;13:1766451. doi: 10.3389/fnut.2026.1766451 (PMC13152728; doi:10.3389/fnut.2026.1766451)
Supplement: Supplementary file 1 [file Table_1.docx]

Supplementary Material

# Supplementary Figures 1


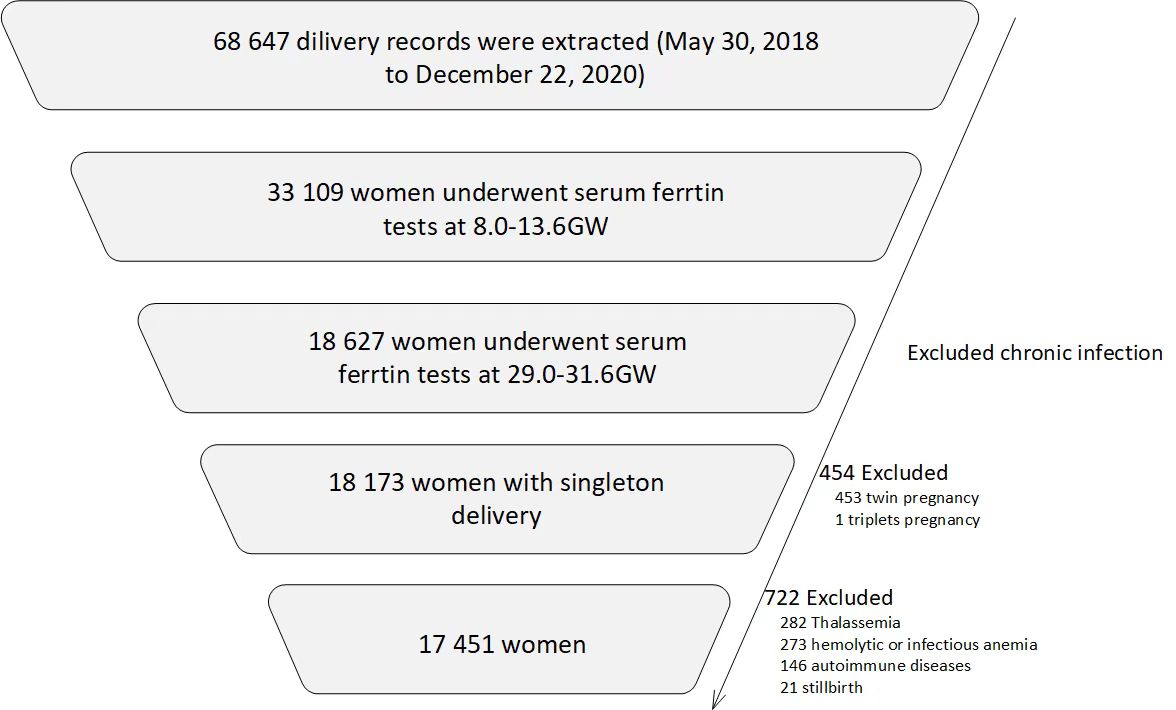


**Supplementary Figure 1.** Flowchart of the study population.
